# A Study on Vitamin B12 Levels in Hypothyroid Patients Presenting to a Tertiary Care Teaching Hospital

**DOI:** 10.7759/cureus.44197

**Published:** 2023-08-27

**Authors:** Ritu Gupta, Sushma Choudhary, Trisha Chatterjee

**Affiliations:** 1 Department of General Medicine, Netaji Subhash Chandra Bose Medical College, Jabalpur, IND

**Keywords:** anti-tpo, anemia, autoimmune, hypothyroid, vitamin b12

## Abstract

Background and aims

The prevalence of vitamin B12 deficiency is found to coexist in hypothyroid patients, causing the persistence of symptoms concomitant to both diseases even on adequate thyroxine supplementation.

Primary objective

To study vitamin B12 levels in patients with hypothyroidism.

Secondary objective

To study the clinical profile of patients with hypothyroidism with special reference to anemia, and to study the association between vitamin B12 deficiency with anti-thyroid peroxidase (anti-TPO) antibodies and anti-thyroglobulin (anti-Tg) antibodies in patients with hypothyroidism.

Methods and results

A single-centric cross-sectional study was carried out over a period of one year. Among 100 hypothyroid patients, 68% were found to be vitamin B12 deficient, among whom 73.5% were females. Of patients with raised anti-TPO antibodies, 78.6% had vitamin B12 deficiency (p = 0.01), while 78% of patients with raised anti-Tg antibodies were vitamin B12 deficient (p = 0.07). The Pearson correlation coefficient (r) of vitamin B12 with anti-TPO and anti-Tg antibodies was -0.302 (p = 0.002) and -0.253 (p = 0.011), respectively.

Conclusion

There is a predilection of hypothyroid patients toward developing anemia, with vitamin B12 deficiency as a major etiology. This finding can be correlated with the hematopoietic action of thyroid-stimulating hormones as well as autoimmune thyroid disease predisposing to pernicious anemia.

## Introduction

Thyroid hormones stimulate the proliferation of erythrocyte precursors both directly and by the enhancement of erythropoietin production [1]. Thus, different forms of anemia might develop in the course of thyroid dysfunction. Anemia that normalizes in response to thyroxine (T4) replacement, even in the presence of normal serum iron, vitamin B12, and folate, is found in up to 25% of hypothyroid patients [2].

Vitamin B12 deficiency is often found to be coexisting in patients with hypothyroidism. The association between autoimmune thyroid disorders and vitamin B12 deficiency is likely due to the concomitant presence of other autoimmune disorders like atrophic gastritis and/or pernicious anemia, both of which result in impaired absorption of vitamin B12 [3]. The number of cases of vitamin B12 deficiency in hypothyroid patients increases with age [4]. Patients with a deficiency of vitamin B12 and hypothyroidism usually have symptoms of fatigue, weakness, poor memory retention, itching, and loss of sensation [5,6], symptoms that are characteristic of both individual diseases.

## Materials and methods

Study center and target population

The present study was carried out at Netaji Subhash Chandra Bose Medical College, Jabalpur, Madhya Pradesh, a tertiary care teaching hospital in central India.

Sample size and study design

The calculated sample size was 95. The study thereby incorporated 100 participants for further evaluation and analysis. This was a single-centric cross-sectional study.

Aims and objectives

Primary Objective

To study vitamin B12 levels in patients with hypothyroidism.

Secondary Objective

To study the clinical profile of patients with hypothyroidism with special reference to anemia, and to study the association between vitamin B12 deficiency with anti-thyroid peroxidase (anti-TPO) antibodies and anti-thyroglobulin (anti-Tg) antibodies in patients with hypothyroidism.

Inclusion and exclusion criteria

The inclusion and exclusion criteria are mentioned in Table 1.

**Table 1:** Inclusion and exclusion criteria

Inclusion criteria	Exclusion criteria
Known cases of hypothyroid patients admitted under the department of medicine	Patients having a vegan diet
Newly diagnosed hypothyroid patients	Patients having a history of gastric or ileal resection
Cases of both subclinical and overt hypothyroidism	Patients having a history of pancreatic insufficiency
Age ≥ 14 years	Patients with a history of malabsorption syndrome
	Patients with chronic diseases like chronic kidney disease, chronic liver disease, rheumatoid arthritis, and systemic lupus erythematosus, which may lead to anemia
	Alcoholic subjects with a history of alcoholism for ≥1 year
	Patients chronically on drugs known to interfere with vitamin B12 absorption
	Patients on vitamin B12 supplementation
	Known cases of vitamin B12 deficiency of other etiology
	Patients in their antepartum period and puerperium

Study definitions

In this study, vitamin B12 deficiency will be defined as serum vitamin B12 < 160 pg/mL, and folate deficiency will be defined as serum folic acid < 2 ng/mL.

Techniques

Patients on admission were subjected to history taking and thorough general, head-to-toe, and systemic examination. Subsequently, 2 mL venous blood samples were collected and sent in ethylenediaminetetraacetic acid (EDTA) vials for complete blood count and peripheral smear studies for each subject. Measurement of serum iron was done by colorimetric method, whereas total iron-binding capacity (TIBC) was determined using spectrophotometry. Serum concentrations of free triiodothyronine (FT3), free thyroxine (FT4), thyroid-stimulating hormone (TSH), vitamin B12, folate, and ferritin were measured using the chemiluminescence immunosorbent assay. Thyroid autoantibodies (anti-TPO and anti-Tg antibodies) were measured using the enzyme immunoassay method.

Ethical issues

This study was conducted after getting informed written consent from the patients in their local language. The study was approved by the Institutional Ethics Committee, Netaji Subhash Chandra Bose Medical College, Jabalpur, Madhya Pradesh, India (Approval No.: IEC/2020/133).

Data analysis plan

Statistical analyses were performed using Statistical Package for the Social Sciences (SPSS) version 28 software (IBM Corp., Armonk, NY). Descriptive analyses were used for variables in groups. The chi-square test, Student's t-test, and ANOVA test were applied for the determination of the relation between the variables. The Pearson correlation test was used to study the correlation between parametric variables. P-values < 0.05 were accepted as statistically significant.

## Results

This study included 100 hypothyroid patients, and serum vitamin B12 levels were investigated and correlated with the presence of thyroid autoantibodies. In addition, the clinical profile of hypothyroid patients was explored and a comparative analysis of the prevalence of anemia was done.

**Figure 1:**
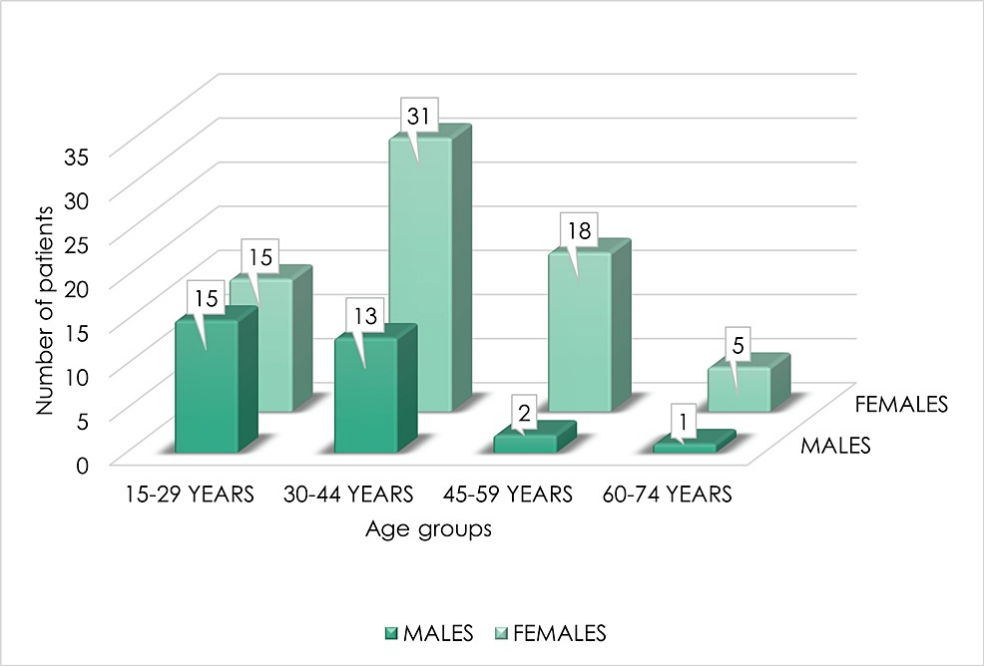
Age and gender distribution of hypothyroid patients

Among the 100 hypothyroid candidates undertaking the study, 31% were males and 69% were females. Of the patients, 44% belonged to the age group of 30-44 years (Table 2 and Figure 1).

**Table 2:** Age and gender distribution of hypothyroid patients

Age group	Males	Females	Total
15-29 years	15	15	30 (30%)
30-44 years	13	31	44 (44%)
45-59 years	2	18	20 (20.6%)
60-74 years	1	5	6 (6%)
	31 (31%)	69 (69%)	n = 100

**Figure 2:**
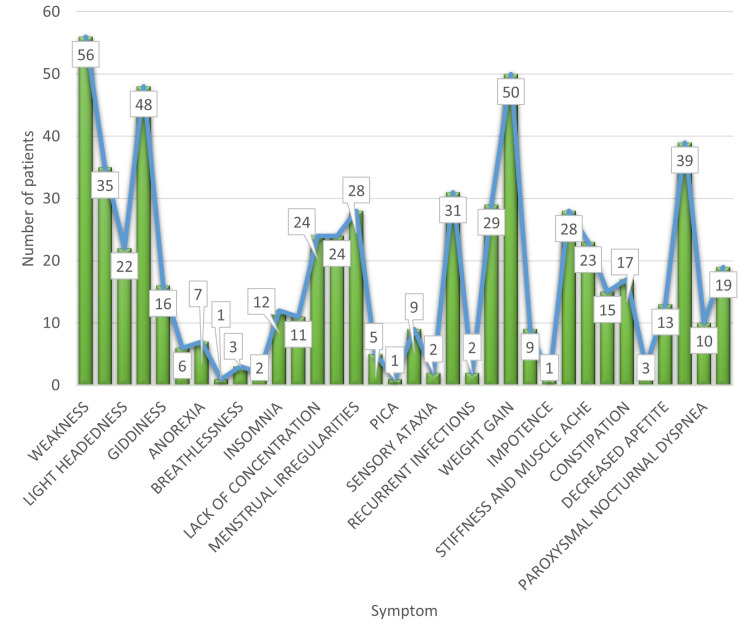
Presenting symptoms of hypothyroidism

**Figure 3:**
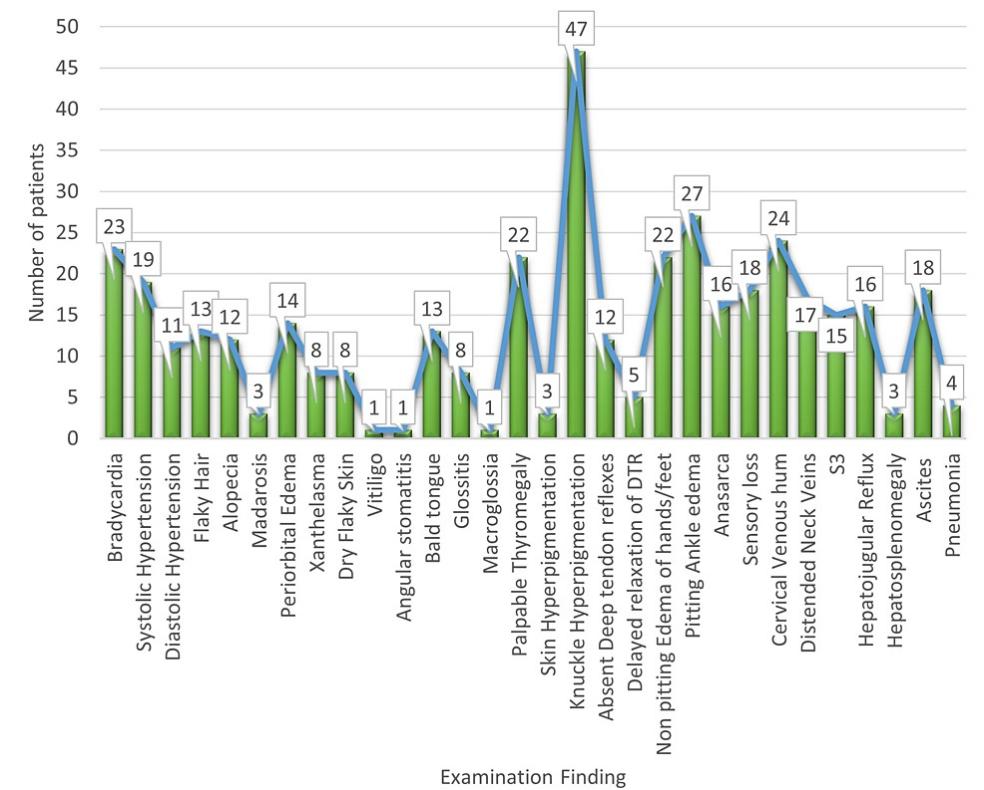
Examination findings of hypothyroid patients S3: third heart sound; DTR: deep tendon reflexes.

The most common presenting complaint in the hypothyroid population was weakness (56%), followed by weight gain (50%) (Figure 2). On examination, 47% of patients had knuckle hyperpigmentation, a sign of vitamin B12 deficiency (Figure 3).

A total of 24% of hypothyroid patients in this study had moderate anemia, with 42% of patients suffering from severe anemia (p = 0.29). Among males, 29% of patients had moderate anemia, while 35% of patients had severe anemia. There was a similar finding among females, out of whom 22% were suffering from moderate anemia, while 45% of patients had severe anemia (Table 3). The mean hemoglobin in the study was 9.45 ± 3.2 g/dL. Of hypothyroid patients included in the study, 53% had a macrocytic blood picture, signifying a probable cause of vitamin B12 deficiency. Of patients, 32% had hyperchromasia, while 31% were normochromic.

**Table 3:** Anemia prevalence in hypothyroid patients

	Males		Females	
	Number	% of males	Number	% of females
Normal hemoglobin (males: >13g/dL; females: >12 g/dL)	8	26	20	29
Mild anemia (males: 11-12.9 g/dL; females: 11-11.9 g/dL)	3	10	3	4
Moderate anemia (8-10.9 g/dL)	9	29	15	22
Severe anemia (<8 g/dL)	11	35	31	45

On analyzing vitamin B12 levels in the hypothyroid population, a total of 68% of patients were vitamin B12 deficient, among whom 42.6% belonged to the age group of 30-44 years. A total of 73.5% of patients who were vitamin B12 deficient were females, which could imply a possible cause of pernicious anemia being prevalent in higher numbers among females.

Of patients, 56% had raised anti-TPO antibody levels, with maximum patients, i.e., 64.3%, with raised antibody titers belonging to the age group of 15-44 years, and 75% were females. The mean anti-TPO antibody level was 89.51 IU/mL. Of hypothyroid patients, 41% had raised anti-Tg antibodies. Most of the patients with raised anti-Tg antibody titers (65.8%) belonged to the age group of 15-44 years, with 65.9% females. The mean anti-Tg antibody level in this study was 123.06 ± 90.13 IU/mL.

**Figure 4:**
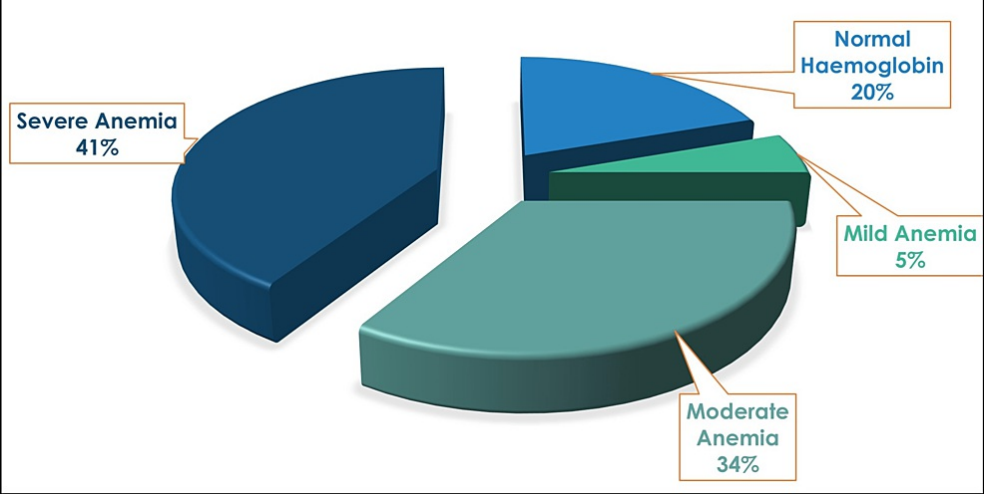
Anemia in patients with raised anti-thyroid peroxidase antibodies

On analyzing a correlation between the prevalence of raised titers of thyroid autoantibodies in the hypothyroid population under study with the severity of anemia, it was found that 33.9% of hypothyroid patients with raised anti-TPO antibody titers had moderate anemia, while 41.1% of patients with raised anti-TPO antibody titers were having severe anemia (p = 0.036) (Figure 4).

**Figure 5:**
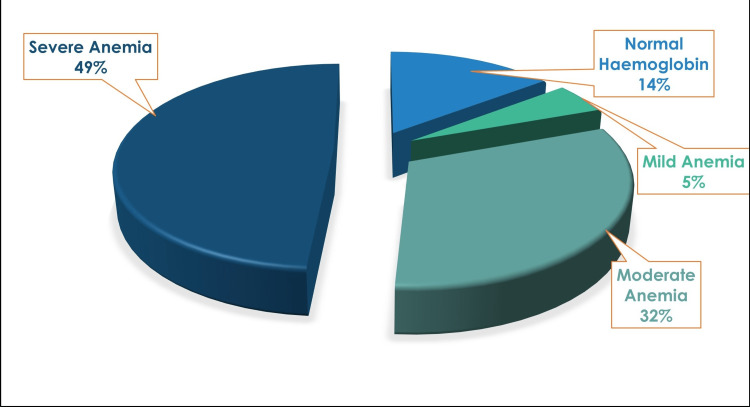
Anemia in patients with raised anti-thyroglobulin antibodies

A similar finding was noted among patients with raised anti-Tg antibody titers. It was observed that 31.7% of hypothyroid patients with raised anti-Tg antibody titers had moderate anemia, while 48.8% of patients with raised anti-Tg antibody titers had severe anemia (p = 0.07) (Figure 5).

**Figure 6:**
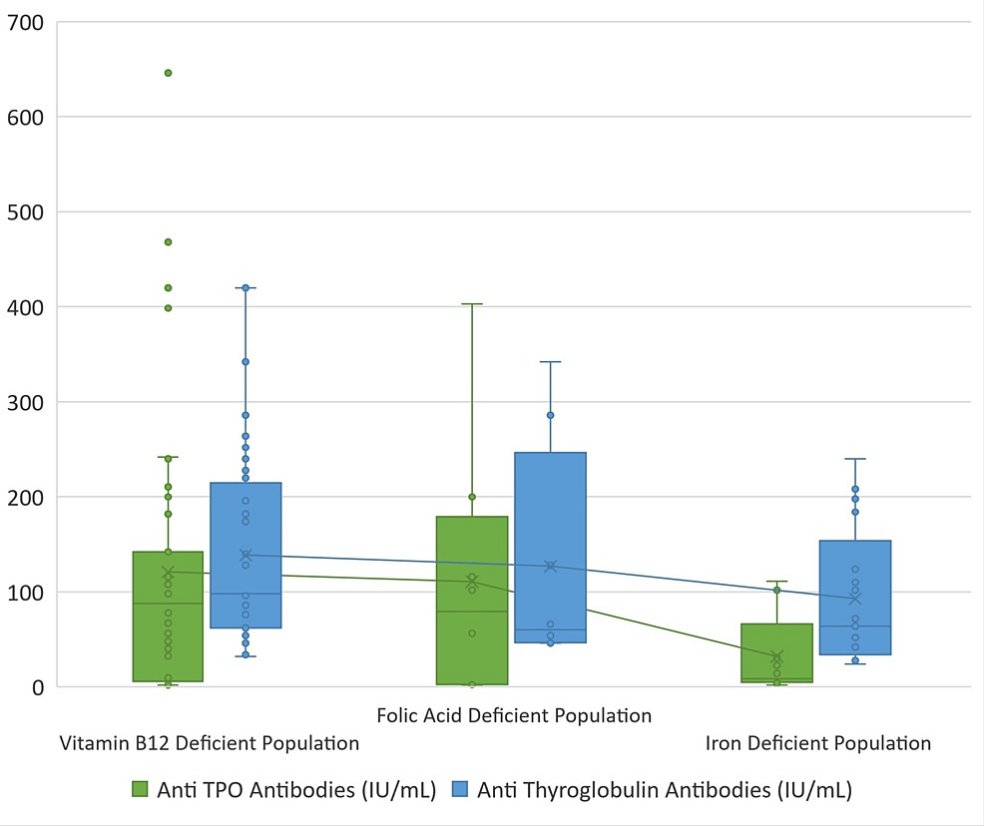
Comparison of the distribution of serum autoantibodies in vitamin B12, folic acid, and iron-deficient population Anti-TPO: anti-thyroid peroxidase.

On correlating vitamin B12 levels with thyroid autoantibodies (Figure 6), 78.6% of patients with raised anti-TPO antibodies were found to have vitamin B12 deficiency (p = 0.01) (Table 4), while 78% of patients with raised anti-Tg antibodies had vitamin B12 deficiency (p = 0.07) (Table 5).

**Table 4:** Vitamin B12 levels correlated with anti-TPO antibodies Anti-TPO: anti-thyroid peroxidase.

	Normal vitamin B12 (>160 ng/L)	%	Vitamin B12 deficiency (<160 ng/L)	%
Normal anti-TPO antibodies (<9IU/mL)	20	45.5% of patients with normal anti-TPO antibodies	24	54.5% of patients with normal anti-TPO antibodies
Raised anti-TPO antibodies (>9IU/mL)	12	21.4% of patients with raised anti-TPO antibodies	44	78.6% of patients with raised anti-TPO antibodies
Total	32%		68%	
p-value = 0.01				

**Table 5:** Vitamin B12 levels correlated with anti-thyroglobulin antibodies Anti-Tg: anti-thyroglobulin.

	Normal vitamin B12 (>160 ng/L)	%	Vitamin B12 deficiency (<160 ng/L)	%
Normal anti-Tg antibodies (<116 IU/mL)	23	39.0% of patients with normal anti-Tg antibodies	36	61.0% of patients with normal anti-Tg antibodies
Raised anti-Tg antibodies (>116 IU/mL)	9	22.0% of patients with raised anti-Tg antibodies	32	78.0% of patients with raised anti-Tg antibodies
Total	32 (32% of total)		68 (68% of total)	
p-value = 0.07				

**Figure 7:**
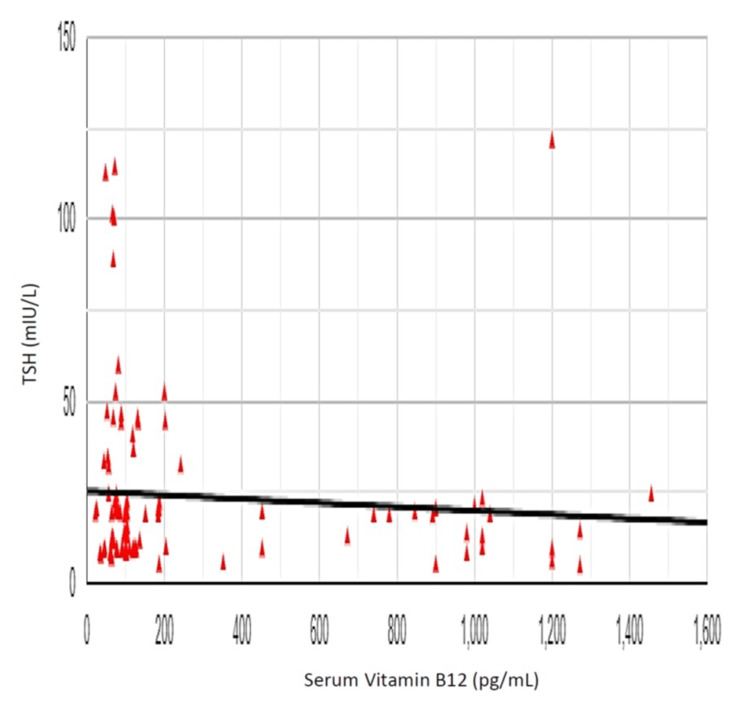
Correlation of vitamin B12 and thyroid-stimulating hormone (TSH)

**Figure 8:**
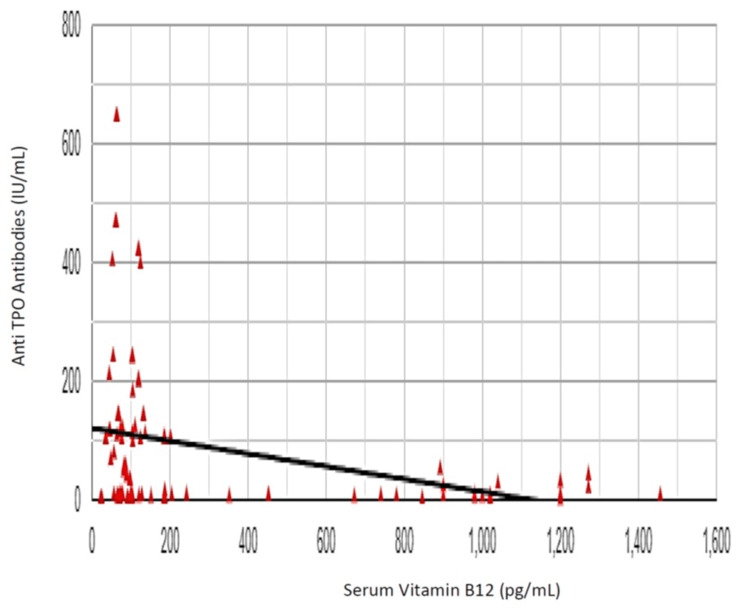
Correlation of vitamin B12 and anti-thyroid peroxidase (anti-TPO) antibodies

**Figure 9:**
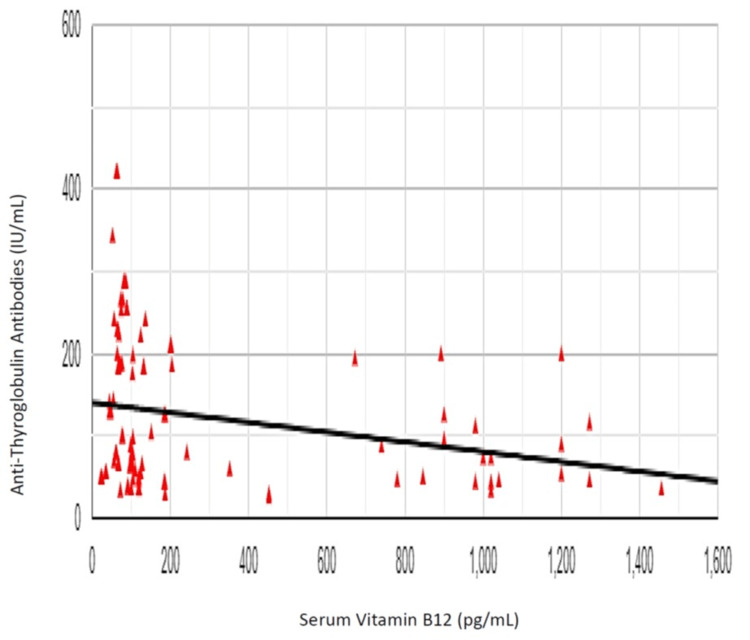
Correlation of vitamin B12 and anti-thyroglobulin antibody levels

The Pearson correlation coefficient (r) between vitamin B12 and TSH was -0.084, showing a weak negative correlation between vitamin B12 and TSH (r (98) = 0.084, p = 0.406) (Figure 7). The Pearson correlation coefficient (r) between vitamin B12 and anti-TPO antibody was -0.302, showing a negative correlation between vitamin B12 and anti-TPO antibody levels (r (98) = 0.302, p = 0.002) (Figure 8). The Pearson correlation coefficient (r) between vitamin B12 and anti-Tg antibody was -0.253, showing a negative correlation between vitamin B12 and anti-Tg antibody levels (r (98) = 0.253, p = 0.011) (Figure 9).

## Discussion

Out of 100 hypothyroid patients included in the study, 31% were males and 69% were females, showing an increased prevalence of the disease in the female population. This was in coherence with a study that included 133 hypothyroid patients, where Aon et al. [7] described 82% of the studied population being females.

The hypothyroid population studied here presented to the hospital with predominant complaints of weakness (56%), followed by weight gain (50%). The most common presenting complaint of hypothyroid patients described by El-Shafie [8] was fatigue, which accounted for 25% of cases, followed by constipation (20%). A stark difference in this study was that a significant proportion of hypothyroid patients, around 38%, were asymptomatic. Another study [9] enumerated the most common chief complaints of hypothyroid patients as lethargy, constipation, cold intolerance, intellectual and motor dullness, dry skin, and hoarse voice, with similar findings in our study.

Antonijević et al. [10] stated that anemia is often the first sign of hypothyroidism, which can be microcytic, macrocytic, or normocytic with regular frequency. Any case of anemia with uncertain etiology should thus raise a clinical suspicion toward the diagnosis of hypothyroidism because signs of overt hypothyroidism might not necessarily be evident. Horton et al. [11] described 202 patients with hypothyroidism having anemia on presentation in 39 of 172 women and 14 of 30 men. The mean corpuscular volume (MCV) exceeded 90 fl in 29 of these 53 patients and in three patients, it was greater than 100 fL, thus describing the macrocytic anemia of hypothyroidism. In our study, the mean MCV was 97.71 ± 16.89 fL, with a maximum value noted at 132.4 fL, which was suggestive of the prevalence of macrocytic anemia in the hypothyroid population, similar to the evidence in the literature.

Of patients in the study, 68% were vitamin B12 deficient, among whom 73.5% of patients who were vitamin B12 deficient were females, also inferring the increased prevalence of vitamin B12 deficiency in the female gender. Jabbar et al. [12] described 39.6% of hypothyroid patients having low serum vitamin B12 levels. The findings in our study were also consistent with those described by Aon et al. [7].

Of patients, 56% had raised anti-TPO antibody levels, while 41% of hypothyroid patients had raised anti-Tg antibodies. In accordance with our study, Jabbar et al. [12] described that 67% of hypothyroid patients had positive titers for anti-TPO antibodies.

We found that 64.7% of patients with vitamin B12 deficiency had raised anti-TPO antibodies. In other words, 78.6% of patients in our study with raised anti-TPO antibodies were found to have vitamin B12 deficiency (p = 0.01). This can explain the nature of vitamin B12 deficiency in the background of autoimmune hypothyroidism. Ness-Abramof et al. [13] described a similar finding of prevalence of vitamin B12 deficiency in 28% of patients having autoimmune hypothyroidism. However, Jabbar et al. [12] described that the prevalence of vitamin B12 deficiency did not differ in patients with positive antibodies (43.2%) compared to those with negative antibodies (38.9%) (p = 0.759).

As a conclusive analysis in our study, the Pearson correlation coefficient (r) correlating vitamin B12 with anti-TPO antibody levels was -0.302, showing a negative correlation between vitamin B12 and anti-TPO antibody levels (r (98) = 0.302, p = 0.002), i.e., a rising anti-TPO antibody level with declining serum vitamin B12 levels. Comparable results were described by Kacharava et al. [14], stating that vitamin B12 levels were inversely correlated to anti-TPO levels (r = -0.233, p < 0.001).

Similarly, the Pearson correlation coefficient (r) correlating vitamin B12 with anti-Tg antibody levels was -0.253, showing a negative correlation between vitamin B12 and anti-Tg antibody levels (r (98) = 0.253, p = 0.011), implying once again, increasing prevalence of vitamin B12 deficiency in patients having raised levels of anti-Tg antibodies.

On the other hand, the Pearson correlation coefficient (r) between TSH and serum vitamin B12 was -0.084, showing a small negative correlation between vitamin B12 and TSH (r (98) = 0.084, p = 0.406), which was, however, statistically non-significant. Analogous to our findings, Aktaş et al. [15] showed that there was a weak, negative, but statistically significant correlation between anti-TPO and vitamin B12 levels in patients with hypothyroidism (r = -0.394, p < 0.001). This shows that TSH, a major factor impacting erythropoiesis, has, however, little effect on vitamin B12. Whereas, we find a significant correlation between vitamin B12 deficiency with thyroid autoantibodies in the serum of hypothyroid patients.

Taken together, the results of our study on a general population of outpatients and inpatients of hypothyroidism prove to be consistent with our hypothesis based on evidence-based literature, stating that there is a predilection of hypothyroid patients toward developing anemia, with vitamin B12 deficiency as a major etiology. Therefore, megaloblastic anemia being present in patients with hypothyroidism, the clinical manifestations and severity of hypothyroidism can have a balanced input from both hypothyroidism and a deficiency of vitamin B12. This conclusive outcome may prove to be of benefit in the thorough evaluation and therapeutic approach to a patient with hypothyroidism.

Limitations of the study

The period of study conducted was of a finite duration, restricting the number of subjects included in the study. In addition, as the study population was restricted to patients presenting to a tertiary healthcare center, the findings may not be suitably applicable or generalized to the whole population. Furthermore, we could not advance with investigations that could direct further to the cause of vitamin B12 deficiency or could accurately predict the autoimmune nature of the disease, like intrinsic factor antibodies or anti-parietal cell antibodies, due to the limitation of resources. This could provide scope for future research in this field.

## Conclusions

Thyroid hormones, as we know, play a major role in the stimulation of erythrocyte precursors; thereby, hypothyroidism is being found to coexist with anemia. Also, an autoimmune etiology of hypothyroidism predisposes to the development of other autoimmune illnesses, and thus should always be sought for once autoimmune thyroid disease is established or suspected in an individual. The correlation of raised thyroid autoantibody levels with vitamin B12 deficiency was increasingly observed in the female population, thus deducing an important association of autoimmune diseases with the female gender.

Clinical manifestations of both hypothyroidism and vitamin B12 deficiency are interlinked, with a lot of signs and symptoms overlapping between the two diseases. It has been observed that even after adequate supplementation of exogenous thyroxine, symptom alleviation may not be achieved in a hypothyroid individual. In such cases, supplementation of vitamin B12 in adequate doses in hypothyroid patients might help in mitigation of symptoms and decrease in severity of the disease. This can be a significant and novel implementation in the routine prescription of hypothyroid patients, aiding in decreasing morbidity and contributing toward the betterment of health and society.
